# A New Extensively Characterised Conditionally Immortal Muscle Cell-Line for Investigating Therapeutic Strategies in Muscular Dystrophies

**DOI:** 10.1371/journal.pone.0024826

**Published:** 2011-09-14

**Authors:** Sofia Muses, Jennifer E. Morgan, Dominic J. Wells

**Affiliations:** 1 Royal Veterinary College, London, United Kingdom; 2 UCL Institute of Child Health, London, United Kingdom; Wellcome Trust Centre for Stem Cell Research, United Kingdom

## Abstract

A new conditionally immortal satellite cell-derived cell-line, H2K 2B4, was generated from the H2K^b^-*ts*A58 immortomouse. Under permissive conditions H2K 2B4 cells terminally differentiate *in vitro* to form uniform myotubes with a myogenic protein profile comparable with freshly isolated satellite cells. Following engraftment into immunodeficient dystrophin-deficient mice, H2K 2B4 cells regenerated host muscle with donor derived myofibres that persisted for at least 24 weeks, without forming tumours. These cells were readily transfectable using both retrovirus and the non-viral transfection methods and importantly upon transplantation, were able to reconstitute the satellite cell niche with functional donor derived satellite cells. Finally using the Class II DNA transposon, *Sleeping Beauty*, we successfully integrated a reporter plasmid into the genome of H2K 2B4 cells without hindering the myogenic differentiation. Overall, these data suggest that H2K 2B4 cells represent a readily transfectable stable cell-line in which to investigate future stem cell based therapies for muscle disease.

## Introduction

Primary myoblasts are routinely used as a cell model for muscle research. However, their limited proliferation capacity *in vitro* hinders their ability to be used in repeated experiments and consequently numerous isolations of primary cells are required. Whilst whole muscle digestion approaches are feasible, the time consuming nature of these methods makes them less than ideal. More importantly, myogenic heterogeneity within every extraction may give rise to conflicting results between experiments. An alternative approach is the use of established muscle cell-lines which have extensive mitotic capacity whilst retaining their ability to terminally differentiate *in vitro* and *in vivo*. The C2 sub-clone, C2C12, is widely used, however, as the cell-line form tumours when transplanted into muscle the cell model is limited to *in vitro* based experiments [1], [2], [3]. In addition, studies conducting 2- and 3 dimensional cell culture experiments have shown differences in myotube maturation as well as myogenic and adhesion protein expression in C2C12 cells compared to primary myoblasts, highlighting the importance of using a suitable cell-line when investigating myogenic differentiation *in vitro*. [4], [5], [6], [7]. In 1994, Morgan *et al*. generated conditionally immortal myogenic cell-lines from the *H-2K^b^*-tsA58 immortomouse [8], [9]. This transgenic mouse harbours a temperature sensitive immortalising T-antigen gene (*ts*A58) under the control of a gamma interferon inducible MHC Class I promoter. Depending on culture conditions, the cells either exhibit continuous mitosis or alternatively terminally differentiate into myotubes. The conditionally immortal nature of the myogenic cells have made them a valuable tool for fundamental myogenic research as well as a suitable cell model for screening drug compounds as potential treatment for Duchenne muscular dystrophy [2], [10], [11], [12], [13]. Since 1994, conditionally immortal myoblasts have been routinely used as cell models for muscle research; however extensive *in vitro* and *in vivo* characterisation on individual cell-lines has yet to be conducted. In addition, as previous conditionally immortal cell-lines have been generated from proliferating myogenic cells and not individual satellite cells, it is possible that the commonly used cell models do not exhibit all of the muscle stem cell qualities [8]. Whilst several studies have used conditionally immortal satellite cell derived cell-lines to address particular hypothesis, the clones were not extensively characterised *in vitro* prior to their used *in vivo* [11], [12].

Herein we described a new conditionally immortal satellite cell derived cell-line, H2K 2B4, which was generated from the *H-2K^b^*-tsA58 immortomouse. The H2K 2B4 clone was extensively characterised *in vitro* and *in vivo* and was shown to exhibit muscle stem cell characteristics in both environments. The myogenic clone was readily transfectable using retrovirus and the non-viral transfection method, Nucleofection, which utilises electrical pulses to introduce exogenous genes. Furthermore, using the Class II transposon, *Sleeping Beauty*, we stably integrated a reporter plasmid into the genome of the H2K 2B4 cells. Through analysing integration sites, the *Sleeping Beauty* transposon exhibited a random integration profile and did not hinder the proliferative or myogenic potential of the H2K 2B4 clone, thus making it a safer alternative to the use of integrating viruses in the generation of genetically modified cell-lines. Overall, H2K 2B4 cell-line has proven to be a reliable “stem cell model” that is ideal for myogenic research.

## Materials and Methods

### Isolating and culturing satellite cells from myofibres

Single myofibres were prepared from extensor digitorum longus (EDL) muscles of a 3 week old male *H-2K^b^*-tsA58 immortomouse as previously described [14]. Single satellite cells were cultured in Matrigel coated 6-well plates (0.1 mg/mL) growth medium [DMEM, 20% (v/v) heat inactivated-foetal bovine serum, 2% (v/v) chicken embryo extract (CEE, Sera laboratories international), 4 mM L-glutamine and 1% (v/v) penicillin/streptomycin]. Matrigel (B.D. Bioscience) was used to encourage cell adhesion. Briefly, wells were coated in 0.1 mg/mL Matrigel (diluted in DMEM), incubated for 30 minutes at 37°C before removing any excess Matrigel. To initiate expression of the thermolabile T-antigen protein, growth media was supplemented with 20 U/mL of γ-IFN (Chemicon) and the cells cultured at 33°C/10% CO_2_.

### Evaluation of terminal differentiation of H2K 2B4 cells *in vitro*

Myoblasts were cultured in differentiation medium [DMEM, 5% (v/v) Horse Serum (PAA Laboratories), 4 mM L-glutamine and 1% penicillin/streptomycin] at a seeding density of 5×10^4^ myoblasts per well in a volume of 250 µl (LABTEK eight-well chamber slides, Nunc). Prior to seeding, wells were coated in 0.1 mg/mL Matrigel, as described above. Myoblasts were incubated for three days at 37°C, 5% CO_2_ without γ-IFN. The medium was not changed as this tended to disrupt myotube formation. H2K 2B4 myoblasts and myotubes were fixed with 4% paraformaldehyde (PFA, Sigma), permeabilised with 0.5% Triton (Sigma) and blocked with 10% goat serum (DakoCytomation). Myoblasts and myotubes were incubated with primary antibodies directed to the following proteins, Pax7 (developmental studies hybridoma bank, DSHB), MyoD (5.8A, DAKO), myogenin (F5D, DSHB), desmin (D33, DAKO) fast and slow myosin (MF20, DSHB) and T-antigen (EMDA Biosciences). Primary antibodies were detected with an appropriate fluorescent secondary antibody. Nuclei were counterstained with DAPI (100 ng/mL, Invitrogen).

### *In vivo* characterisation of the H2K 2B4 cell-line

#### Ethical statement

All animal experiments were carried out under license from the Home Office (UK) in accordance with The Animals (Scientific Procedures) Act 1986 and with approval from Imperial College ethical review process.

#### Contribution to skeletal muscle regeneration in *mdx nu/nu* mice

Prior to irradiation, mice were anaesthetised with Hypnorm (fentanyl/fluanisone, Janssen animal health, UK) and Hypnovel (midazolam, Roche) at a ratio of 1∶1∶2 in injection grade water. Hindlimbs of mdx nu/nu mice were irradiated with 18 Gy at a dose rate of 0.7 Gy/minute [17]. Three days post irradiation, 2 male and 5 female 3 week old mdx nu/nu mice were anaesthetised with isoflurane (Abbott Laboratories) and injected with 5×105 H2K 2B4 cells (total volume of 4 µL) into each irradiated tibialis anterior (TA) muscle. Post-mortem, the engrafted TA muscles were frozen in isopentane pre-chilled in liquid nitrogen 3 and 24 weeks post transplantation. Dystrophin protein expression was detected on 7 µm transverse muscle sections using the polyclonal antibody P7 at a 1/1000 dilution [15].

#### Contribution of functional donor cells to the satellite cell compartment

To identify donor derived satellite cells in vivo, the H2K 2B4 cell-line was infected with 50 cfu/mL of supernatant from the pMFG nls LacZ retrovirus, and 8 µg/mL of polybrene [12], [16]. Nuclear localising beta-galactosidase activity was assessed with X-gal solution as previously described [17]. Transduced H2K 2B4 cells were sub-cloned to generate H2K SC6, a conditionally immortal cell-line with homogenous beta-galactosidase expression. Half a million H2K SC6 cells were engrafted into irradiated TA muscles of two female 3 week old mdx nu/nu mice (mice were anaesthetised and irradiated as described above). Three weeks post engraftment, TA muscles were removed and myofibres were isolated as previously described (Collins and Zammit, 2009). Isolated myofibres were fixed with 4% PFA, blocked with 10% goat and 10% (v/v) swine serum (DakoCytomation) and immunostained with antibodies towards the beta-galactosidase (Molecular probes) and Pax7 proteins, myonuclei were counterstained with DAPI.

To verify donor derived satellite cells were functional, 5×10^5^ H2K SC6 cells were engrafted into irradiated right and left TA muscles of six 23–25 day old male *mdx nu/nu* mice (mice were anaesthetised and irradiated as above). Twenty-one days later mice were reanaesthetised and right TA muscles were injected with 10 µL of *Notechis scutatus scutatus* notexin (10 µg/mL in PBS, Latoxan, France). Left TA muscles were not treated with notexin. Seven days later both TA muscles were removed and immunostained with antibodies targeting neonatal myosin (BF34, DSHB) and dystrophin (P7) proteins. To confirm myofibres were from donor origin, serial sections were stained with X-gal solution to detect beta-galactosidase activity and hematoxylin and eosin (H&E) to assess muscle morphology. Within notexin treated muscle, myofibres co-expressing neonatal myosin and dystrophin within areas expressing beta-galactosidase were counted. As we used a nuclear localising LacZ gene, we did not count beta-galactosidase myofibres to estimate regeneration efficiency as this would have underestimated the number of donor derived myofibres, as previously described [18], [19].

### Genetically modifying H2K 2B4 cells using non viral transfection methods

#### Optimising non-viral transfection methods

When using Lipofectamine 2000, H2K 2B4 cells were seeded at 1×10^5^ cells/well into six-well plates. The following day the H2K 2B4 myoblasts were transfected with Lipofectamine 2000 according to the manufacturer's instructions. For Nucleofection, 5×10^5^ H2K 2B4 cells were resuspended in 100 µL of Amaxa solution V prior to adding the appropriate amount of plasmid DNA (amount dependant on experiment). Cell suspensions were nucleofected using programme B-032 on the Nucleofector II machine (Lonza). Transient transfection efficiencies were analysed using a BD FACS Calibur flow cytometry 48 hours post transfection. To ensure analysis was conducted on viable cells propidium iodide (1/500 dilution, 1 µg/mL, Invitrogen) was added to samples prior to analysis. For each sample, 10,000 viable cells were counted using the Cell-Quest Programme.

#### Optimising stable integration of the Sleeping Beauty transposon

The reporter plasmid, pMONO-neo-eGFP (originally pMONO-neo-LGFP from Invivogen, LGFP was replaced with eGFP gene), was inserted in between the terminal repeats of the SB transposon plasmid, pT2/BH [20], creating a new transposon called pT2/MONO-neo-eGFP. For the in vitro transposition assay different amounts of a plasmid expressing the hyperactive transposase, SB100 [21], were used to determine the optimal ratio of the SB transposon to SB transposase. Using Nucleofection, 5×105 H2K 2B4 cells were co-transfected with 500 ng of pT2/MONO-neo-eGFP and varying amounts of SB100 transposase plasmid (0, 10, 50, 100, 250, 500 & 1000 ng). Forty-eight hours post transfection, growth medium was replaced with fresh growth media supplemented with 800 µg/mL of G418. Growth media supplemented with 800 µg/mL of G418 was changed every 2–3 days. G418 resistant clones were counted after 14 days of G418 selection.

#### Analysis of Sleeping Beauty transposon integration sites

Genomic DNA (gDNA) was extracted from a mixed population of H2K 2B4 G418 resistant colonies, digested with the SspI enzyme and cleaned within phenol∶chloroform∶isoamyl alcohol prior to ligation. To encourage self ligation, gDNA was ligated using 3 U of ligase (Roche) in a total volume of 500 µl at 22°C overnight. Ligated gDNA was cleaned with phenol∶chloroform∶isoamyl alcohol, precipitated in ethanol and electroporated into 50 µl of ElectroMAX DH10B T1 phage competent bacteria (Invitrogen). Transformed bacteria were grown overnight at 30°C on LB agar plates containing kanamycin (50 µg/mL, Sigma). Kanamycin resistant colonies were counter selected on LB agar plates supplemented with kanamycin and ampicillin (50 µg/mL, Sigma). Plasmid DNA was extracted from Kanresistant and Ampsensitive colonies and integrations sites were analysed using primers that hybridise to the left and right terminal repeats of the transposon (Left ITR: 5′GACTTGTGTCATGCACAAAGTAG and Right ITR: 5′CCACTGGGAATGTGATGAAAG [39]). Sequencing reactions were analysed using a nucleotide basic local alignment search tool (nBLAST) against the NCBI mouse genome database. Of the 90 sequencing reactions analysed, 58% were transposase mediated unique integration sites which were mapped, the remaining were discarded (14% were non-transposase mediated integrations, 14% repeated integration sites of transposase mediated integration and 10% bad sequencing, thus preventing mapping of integration sites).

### Imaging

Immunofluorescent images were captured using a Leica DMR microscope (Leica Microsystems, Germany) interfaced with MetaMorph (Molecular Devices, USA). Images were taken at set exposures when comparing groups. For cell counts, five random fields containing approximately 100 myonuclei were chosen.

### Statistical analysis

All data are presented as means ±SEM. Statistical analysis was performed either by a Mann-Whitney test or a one way ANOVA followed by a post hoc Tukey's multiple comparison test. Statistical significance was defined as a value of p<0.05.

## Results

### Generation of satellite cell derived cell-lines

To assess heterogeneity, doubling times and myogenicity was evaluated for four satellite cell derived cell-lines. All clones had similar doubling times; however a variation in the formation of multinucleated myotubes and the expression of the myogenic proteins Pax7, MyoD, myogenin and slow myosin was observed between the clones (Figure S1a). The H2K 2B4 cell-line was the most myogenic clone as it formed more multinucleated myotubes compared to the other cell-lines and was therefore chosen for further characterisation (Figure S1b).

### Differentiation of the H2K 2B4 cell-line *in vitro*

**Figure 1:**
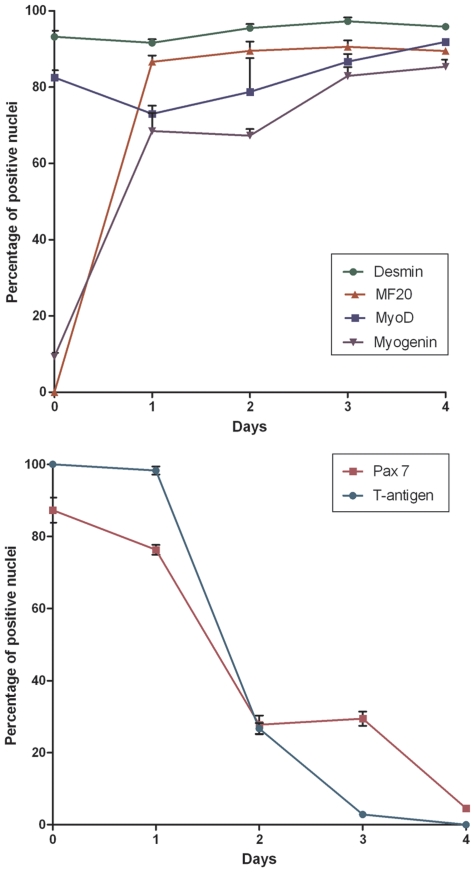
Myogenic protein expression of H2K 2B4 cells during terminal differentiation. H2K 2B4 cells were grown in non-permissive conditions (37°C and no γ-IFN)) over a four day period to assess myogenic protein expression during terminal differentiation *in vitro*. Cells were fixed daily with 4% PFA, at similar time points. Timelines show the variation in protein expression during the 96 hour time period. Error bars were generated from standard error of the mean (SEM). N value of 5 for each protein and time point.

**Figure 2:**
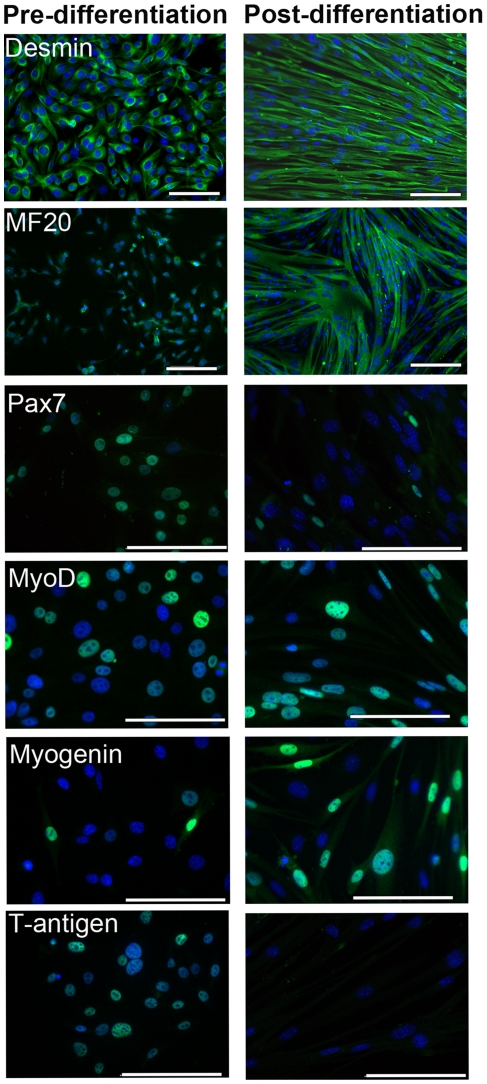
Comparison of myogenic protein expression in H2K 2B4 myoblasts and myotubes. Images comparing protein expression in proliferating H2K 2B4 myoblasts and terminally differentiated myotubes. MF20 antibody targets fast and slow myosin. T-antigen and myogenic proteins are shown in green. Nuclei were counterstained with DAPI (blue). Scale bars: 50 microns.

An *in vitro* differentiation assay was conducted to evaluate myogenic protein expression during terminal differentiation. Over a four day differentiation assay the expression of Pax7, MyoD, myogenin, desmin, fast/slow myosins and the immortalising protein, T-antigen were assessed daily. Myotube formation began within twenty-four hours after switching to differentiation conditions (37°C & no γ-IFN) with the myotube size increasing throughout the assay. At day 0 (cells were fixed once they had adhered to the plate, approximately 3 hours post seeding) over 80% of myoblasts expressed Pax7, a protein associated with quiescent and activated satellite cells, as well as MyoD, a protein required to initiate terminal differentiation (Figure 1). Within the first twenty-four hours myogenin expression was markedly up-regulated and the number of nuclei expressing myogenin increased over the 96 hours, indicating that the majority of myoblasts had committed to terminal differentiation. In contrast to myogenin, cells expressing the Pax7 protein steadily decreased to 5% by 96 hours. Throughout the differentiation assay, myotube maturation coincided with the down-regulation of the thermolabile T-antigen, confirming the immortalising protein did not inhibit the natural myogenic characteristics of the cell-line (Figures 1 and 2). The H2K 2B4 cell-line readily formed multinucleated myotubes even after extensive cell culturing (tested in cells up to 24 continuous passages). Overall, the expression profiles of the myogenic proteins were comparable to previous literature on freshly-isolated satellite cells [22], showing that the H2K 2B4 clone was a good satellite cell model *in vitro*.

### Regeneration of skeletal muscle by the H2K 2B4 clone

**Figure 3:**
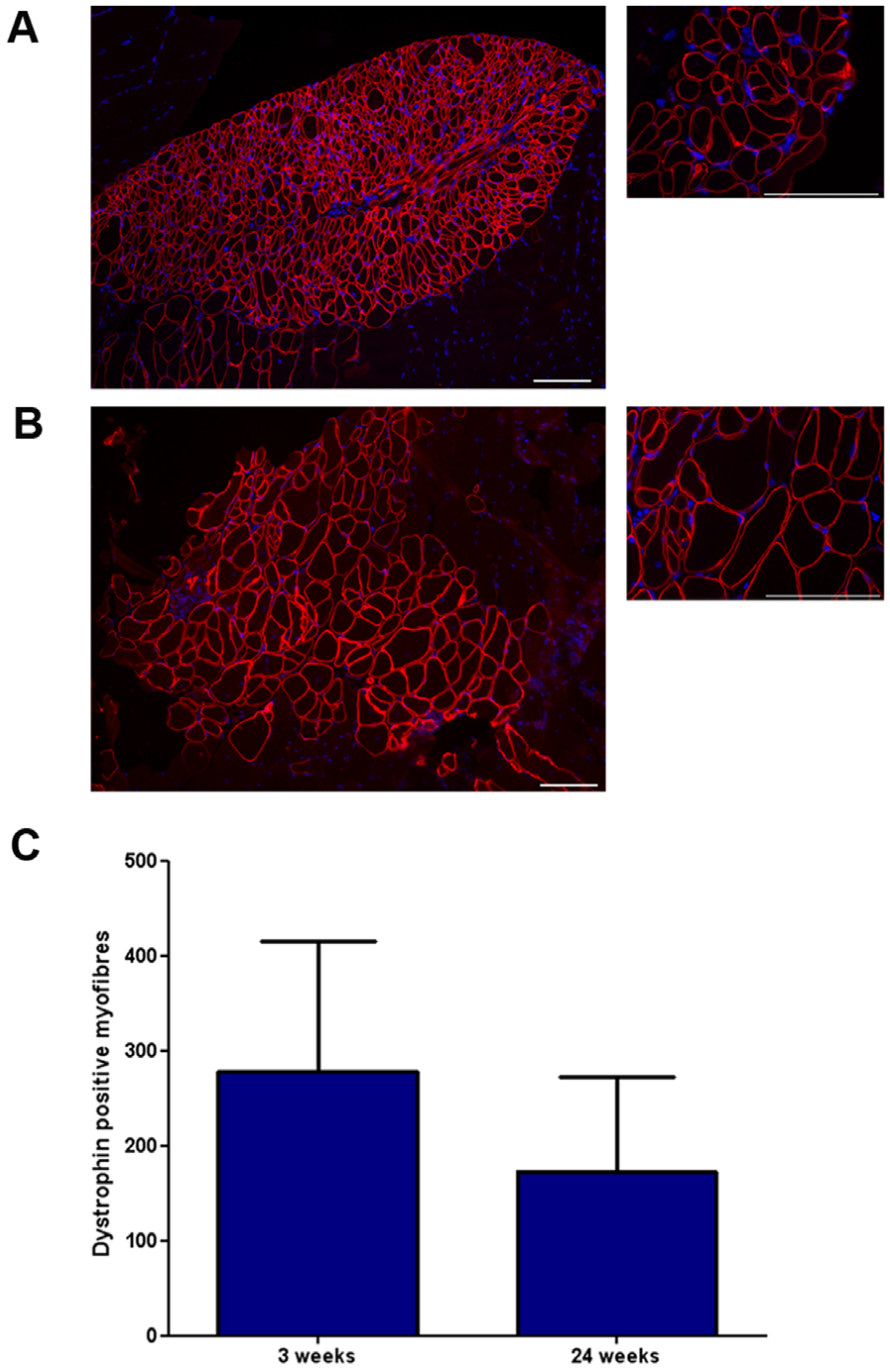
H2K 2B4 cells regenerate muscle *in vivo*. Half a million H2K 2B4 cells were engrafted into irradiated TA muscles of seven *mdx nu/nu* mice. A) Images showing dystrophin positive myofibres (red) derived from donor H2K 2B4 cells three weeks post transplantation and B) twenty-four weeks post transplantation. Nuclei were counterstained with DAPI (blue). Scale bars: 50 microns. C) No significant difference in the number of dystrophin positive myofibres was observed between 3 weeks and twenty-four weeks post transplantation. P>0.05, Mann Whitney test. Error bars were generated from SEM. N value of 8 and 6 mice for 3 weeks and twenty-four weeks post transplantation, respectively.

To determine if the H2K 2B4 cell-line was capable of regenerating skeletal muscle, the cells were engrafted into *mdx nu/nu* mice, a dystrophin deficient mouse model [23], [24]. 5×10^5^ H2K 2B4 cells generated a mean of 279 dystrophin positive myofibres (n = 8, SEM = 137.4) three weeks post engraftment. There was no significant difference in the number of dystrophin positive myofibres 24 weeks post engraftment (mean = 173, n = 6, SEM = 99.15) (Figure 3). More importantly, engrafted muscles were of normal size and morphology indicating the H2K 2B4 cell-line did not form tumours *in vivo* (data not shown).

### H2K 2B4 cells form functional satellite cells *in vivo*

**Figure 4:**
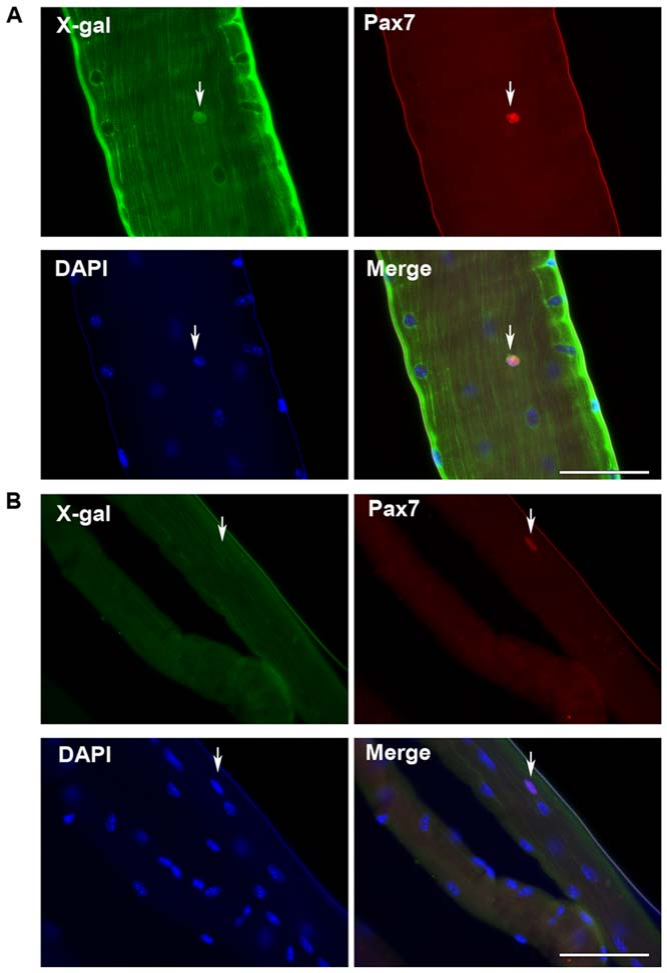
Isolated myofibres from engrafted TA muscles showing H2K SC6 derived satellite cells. H2K SC6 cells were engrafted into irradiated TA muscles of two *mdx nu/nu* mice. Three weeks post transplantation myofibres were isolated and stained for the quiescent maker, Pax7. Donor derived satellite cells were confirmed by the expression of the detection marker beta-galactosidase. A) A H2K SC6 derived satellite cell expressing Pax7 and beta-galactosidase. B) Host “radiation resistant” satellite cell expressing the Pax7, but not beta-galactosidase. Magnification ×40, Scale bars: 50 microns.

**Figure 5:**
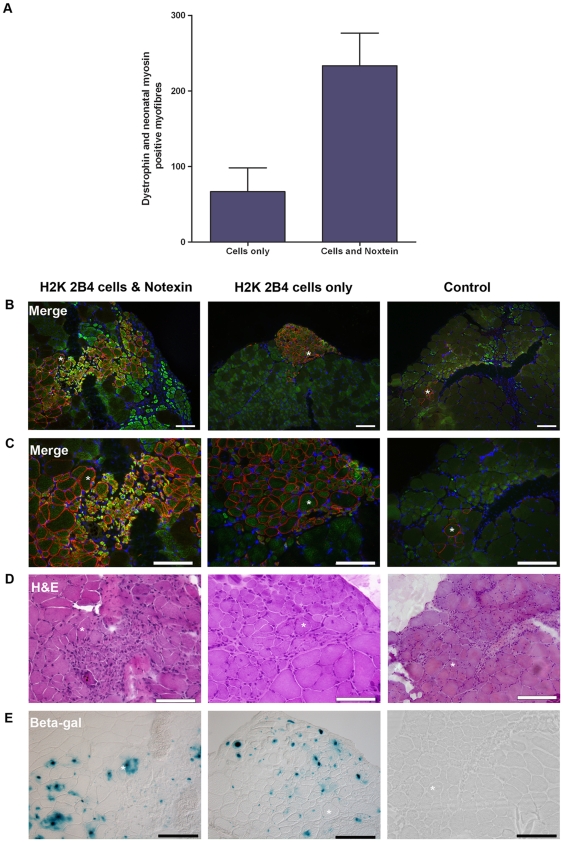
Donor derived H2K SC6 satellite cells give rise to functional satellite cells. Tibialis anterior muscles of six male *mdx nu/nu* mice were irradiated and injected with 5×10^5^ H2K SC6 cells. To determine if H2K SC6 derived satellite cells were functional the right engrafted TA muscle were challenged with notexin three weeks post transplantation. The left engrafted TA muscles were not treated with notexin. Seven days later both TA muscles were removed and H2K SC6 derived satellite cell function was assessed by the number of myofibres co-expressing Dystrophin (Dys) and Neonatal Myosin (NM). A) Engrafted TA muscles treated with notexin had a significant increase in the number of myofibres co-expressing Dys^+^ (red) and NM^+^ (green) positive compared to the non-notexin treated contralateral muscle TA muscle. Myonuclei were counterstained with DAPI (blue). Mann Whitney test, p = 0.0129, N value of 6. Error bars were generated from SEM. B&C) Images showing an increased number of Dys^+^ (red) and NM^+^ (green) myofibres in an engrafted TA muscle treated with notexin, compared to a non-notexin treated engrafted muscle and control muscle (no H2K SC6 cells or notexin). H&E and X-gal staining of serial sections confirmed regenerating myofibres were from donor origin (D&E). Asterisks showing the same myofibre in serial sections. Scale bars: 50 microns.

In addition to regenerating dystrophic muscle, we investigated if H2K 2B4 cells were capable of contributing functional satellite cells to the satellite cell population *in vivo*. In order to distinguish between donor and host satellite cells, we generated a beta-galactosidase expressing satellite cell derived cell-line, H2K SC6, which had been sub-cloned from genetically modified H2K 2B4 cells. Through *in vitro* analysis we confirmed the H2K SC6 clone maintained the myogenic properties of the H2K 2B4 clone. 5×10^5^ H2K SC6 cells were injected into irradiated TA muscles of two female *mdx nu/nu* mice. Myofibres were isolated three weeks post engraftment and stained with antibodies to the satellite cell marker, Pax7 and beta-galactosidase, to identify nuclei of donor origin. We analysed 41 myofibres from 4 engrafted TA muscles and we identified 71 satellite cells, of which 37 (52%) were of donor origin (β-gal and Pax7 positive). Figure 4A shows a representative image of our findings with a nucleus positive for both the Pax7 and beta-galactosidase protein showing that the satellite cell was of donor origin. Pax7 positive/beta-galactosidase negative nuclei was also detected (Figure 4B), which were likely to be to be “radiation resistant” satellite cells [25]. To ensure the donor derived satellite cells were functional an experimentally-induced muscle regeneration study was conducted [17], [18], [26], [27]. 5×10^5^ H2K SC6 cells were injected into each irradiated TA muscle of six male *mdx nu/nu* mice. Three weeks post transplantation, notexin was injected into the right TA muscle. The left TA muscle was not treated with notexin. Seven days later, TA muscles were removed post-mortem and the expression of dystrophin and neonatal myosin was assessed. Both notexin and non-notexin treated muscles contained myofibres co-expressing dystrophin and neonatal myosin. However, the numbers of myofibres co-expressing the two proteins was significantly greater in notexin treated muscles, indicating recent regeneration (n = 6, p = 0.0129) (Figure 5a, b, c). H&E (for muscle morphology) and X-gal (for beta-galactosidase activity) stains confirmed newly-regenerated myofibres were of donor origin, evidence that the H2K SC6 cells were capable of forming functional satellite cells *in vivo* (Figure 5d,e).

### Genetically modifying the H2K 2B4 cell-line

**Figure 6:**
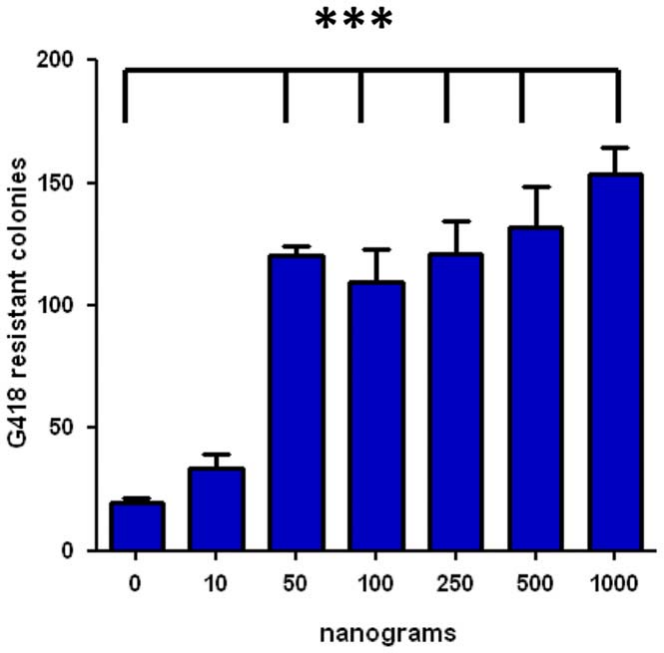
*Sleeping Beauty* transposition assay in the H2K 2B4 cell-line. 500 ng of the *Sleeping Beauty* (*SB*) pT2/MONO-neo-eGFP plasmid and varying amounts of the SB100 transposase plasmid (0, 10, 50, 100, 250, 500, 1000 ng) was co-transfected into the H2K 2B4 cell-line. H2K 2B4 G148 resistant colonies were counted after 14 days of G418 selection. A significant increase in G418 resistant colonies was observed with cells co-transfected with 50,100, 250, 500 and 1000 ng of the SB100 transposase plasmid compared to the cells transfected with the transposon only, suggesting transposase mediated integration had occurred. ^***^ p<0.001. One way ANOVA and Tukey test, N value of four. Error bars generated by SEM.

**Figure 7:**
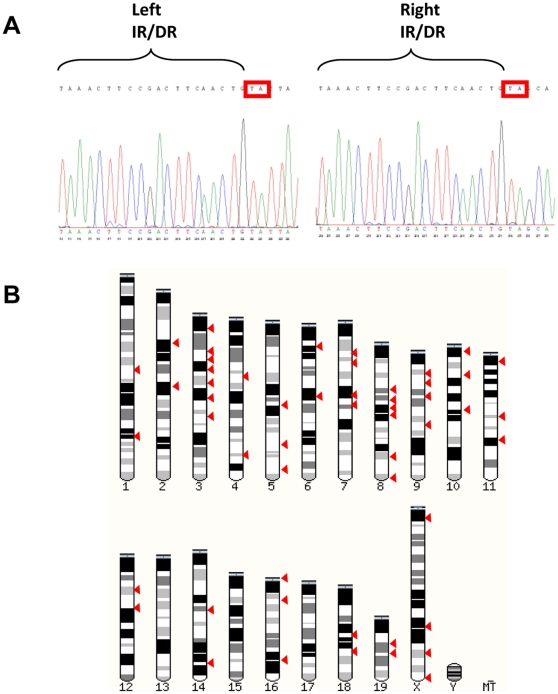
Integration sites of the *Sleeping Beauty* transposon within the genome of H2K 2B4 cells. A) Molecular proof of *SB* transposon integration in the H2K 2B4 cells. Sequencing results showing duplicated TA dinucleotides sites flanking left and right terminal repeat of the *Sleeping Beauty* transposon. B) Schematic diagram showing integration profile of 53 unique SB transposon integration sites within the H2K 2B4 genome.

**Figure 8:**
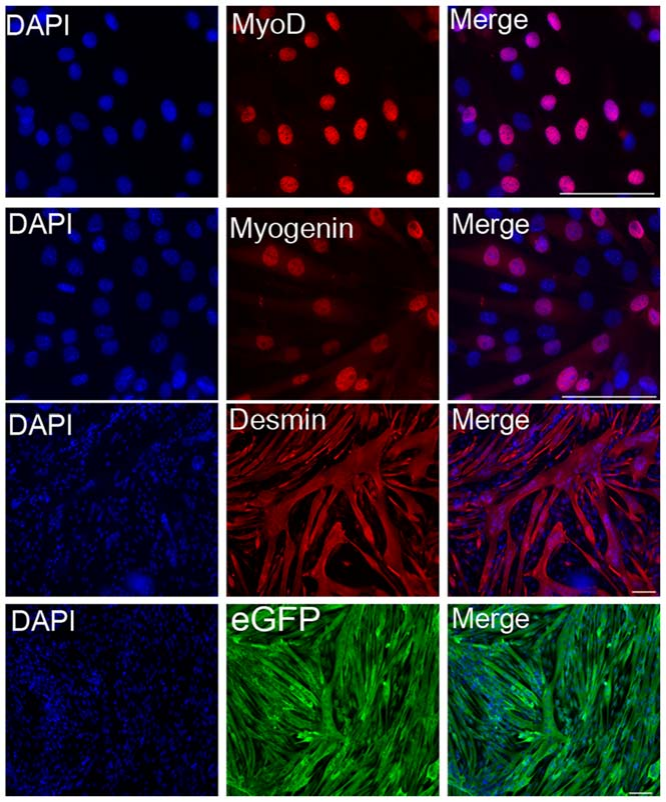
*In vitro* differentiation of H2K 2B4 cells genetically modified with the *Sleeping Beauty* transposon. To determine if genetically modified H2K 2B4 cells were still able to retain their differentiation ability post genetic modification the cell-line was grown in non permissive conditions (37°C and no γ-IFN). Myotubes were immunostained with antibodies towards myogenic proteins (MyoD, myogenin and desmin) and for the transgenic marker, GFP. Scale bars: 50 microns.

We have shown the H2K 2B4 cell-line can be readily transduced with the pMFG nls LacZ retrovirus, an observation consistent with previous conditionally immortal cell-lines [2], [11], [12]. We next investigated if the H2K 2B4 cell-line could be efficiently genetically modified using non-viral transfection methods as limited work on such a system has been conducted to date. Two non-viral transfection methods, Lipofectamine 2000 and Nucleofection, were assessed. Using Lipofectamine, 30% of the H2K 2B4 cells transiently expressed the eGFP protein, whereas, Nucleofection yielded greater transient cell transfection efficiencies of 50–70%. Although not quantified, Nucleofection resulted in a lower cell toxicity compared to Lipofectamine 2000. When using Nucleofection, genetically modified H2K 2B4 cells were able to differentiate into multinucleated myotubes *in vitro* (Figure S2). In addition, the H2K 2B4 clone was readily transfectable even after 27 continuous passages (approximately 80 doublings). Subsequently, we explored the possibility of stably integrating a reporter plasmid into the genome of the H2K 2B4 cells using the *Sleeping Beauty* transfer system [28]. Using Nucleofection, H2K 2B4 cells were co-transfected with the *SB* transposon, pT2/MONO-neo-eGFP, and varying amounts of the hyperactive SB100 transposase plasmid (0, 10, 50, 100, 250, 500 & 1000 ng). Transfected cells were selected with G418 for two weeks and G418 resistant colonies were counted. H2K 2B4 cells transfected with the minimum amount of SB100 plasmid (50 ng), had a significant increase in the number of G418 resistant colonies compared to the negative control (n = 4, p<0.05). A six fold increase in G418 resistant colonies was observed when using 1000 ng of the SB100 transposase plasmid (Figure 6). To confirm the increase in G418 resistant colonies was due to transposase mediated integration rather than non-transposase mediated integration (insertion into the genome without the use of the SB100 transposase), we analysed the integration sites using a plasmid recovery strategy [29]. The plasmid recovery technique distinguishes between non-transposase mediated integration and transposase mediated integration on the basis of antibiotic resistance in bacterial colonies. Colonies which are Kan^resistant^/Amp^resistant^ indicate non-transposase mediated integration, whereas Kan^resistant^/Amp^sensitive^ colonies indicate transposase mediated transposition (Figure S3). Like other Tc1 transposons, the *SB* system has a signature integration profile of targeting TA dinucleotides [30]. Once integrated, the transposon generates TA dinucleotides flanking either side of the terminal repeats, thus allowing the confirmation of transposition events at a molecular level. From the 53 unique integration sites analysed, the integration profile of the *SB* system was random, as it did not target a particular chromosome or gene region (Figure 7), an observation that has been previously reported in other cell types [30], [31], [32], [33]. Of the 53 unique integration sites, 33% were found in active genes, of which all were in introns. Furthermore, the stable integration of the *SB* transposon did not hinder the differentiation ability of the H2K 2B4 cell-line *in vitro* (Figure 8).

## Discussion

Myogenic cells from the *H-2K^b^*-*ts*A58 immortomouse are an ideal cell model system for muscle research, due to extensive mitotic capability and good fusion rate *in vitro* and *in vivo* [8]. Whilst previous studies have used conditionally immortal myogenic cell-lines to address particular hypotheses [10], [11], [12], [17], extensive *in vitro* and *in vivo* characterisation on a particular myogenic cell-line from the *H-2K^b^*-*tsA58* immortomouse has not been conducted. Furthermore, as many previous conditionally immortal cell-lines were derived from enzymatically-disaggregated newborn mouse muscle, they may not be satellite cell derived and could instead represent another stem cell present in skeletal muscle [34]. In light of this, we generated a satellite cell derived clone from the *H-2K^b^*-*ts*A58 immortomouse and assessed its potential to represent a good “muscle stem model” *in vitro* and *in vivo*.

Each conditionally immortal cell-line was generated from a single satellite cell that had migrated away from a myofibre. This was deemed the best isolation method as it ensured the clones were generated from a satellite cell, and prevented contamination from other cell-types present within the muscle. Initial characterisation showed that despite similar doubling times, the ability to form multinucleated myotubes varied between the four cell-lines. It is likely the difference in myogenicity is due to satellite cell heterogeneity [19], [26], [35]. The H2K 2B4 cell-line was chosen for further characterisation as it reliably formed multinucleated myotubes *in vitro* and therefore was deemed the most myogenic clone. Myogenic transcription factors such as Pax7, MyoD and myogenin play an important role in the satellite cell cycle [22], [36]. We therefore analysed their expression levels in the H2K 2B4 cells during myogenic terminal differentiation *in vitro*. Using a robust differentiation assay we confirmed that despite continuous proliferation (approximately 22 doublings), when switched to non-permissive conditions, the H2K 2B4 cell-line exhibited similar myogenic protein expression as seen in freshly isolated satellite cells [22], [37]. The H2K 2B4 cell-line was also capable of forming uniform myotubes after 24 continuous passages (approximately 68 doublings), confirming the cell's myogenicity was not affected by extensive cell culturing. The combination of continuous proliferation whilst retaining a similar terminally differentiating pattern as satellite cells suggests the H2K 2B4 cell-line represents a good “stem cell model” for *in vitro* studies.

When transplanted into irradiated TA muscles of *mdx nu/nu* mice the H2K 2B4 cells regenerated host muscles at similar efficiencies to other conditionally immortal muscle cell-lines [11], [12]. More importantly, the clone was able to form stable myofibres, without forming tumours as seen with C2C12 cells, highlighting the advantage of using *H-2K^b^* derived cell-lines [1], [2]. In addition to regenerating dystrophic muscle, the H2K 2B4 clone was capable of contributing functional satellite cells to the satellite cell niche *in vivo*, an imperative quality which ensures long-term persistence of donor derived cells *in vivo*. Interestingly, during *in vitro* terminally differentiation we identified 5% of H2K 2B4 nuclei expressing Pax7 96 hours after being switched to non-permissive conditions. Therefore, it is possible this subset of Pax7 positive cells is a “stem-cell” population which was responsible for the regeneration and re-occupying the satellite cell niche upon muscle transplantation. Thus, the myogenic protein expression of H2K 2B4 cells is not homogenous and like satellite cells, the H2K 2B4 cells may adopt divergent fates [19], [22].

Overall, in conjunction with the *in vitro* characterisation, the *in vivo* experiments confirmed the H2K 2B4 cell-line exhibited satellite cell qualities making it an ideal cell model for muscle research.

To further validate the H2K 2B4 cell-line as a robust muscle stem cell model we investigated if the cell-line was readily transfectable using viral and non viral techniques. Consistent with previous conditionally immortal cell-lines, the H2K 2B4 cell-line was readily transduced when using the pMFG nls LacZ retrovirus [11], [12]. Conditionally immortal myogenic cells are routinely genetically modified with retroviruses as these hard-to-transfect cells yield low transfection efficiencies when using non viral transfection methods such as Lipofectamine 2000. However, here we have shown the H2K 2B4 cell-line can be efficiently transfected and maintain good cell viabilities when using a less toxic non viral transfection method, Nucleofection. This transfection method combines electrical pulses with specific cell-type solutions to introduce exogenous DNA into the nucleus of target cells. We also showed the cell-line was readily transfectable after extensive cell culturing (tested in cells with up to 80 cell doublings), thus confirming the clone is suitable for long-term experiments. In addition to transient transfection efficiencies, we sought to evaluate stable transgene expression using a non-viral system such as *Sleeping Beauty (SB)*, a class II DNA transposon [28]. Utilising its inherent ability to integrate into host genomes, the *SB* system has been used to introduce reporter and therapeutic genes into a variety of target cells [38], [39], [40], [41], [42]. Here we showed the *SB* system was capable of stably integrating a reporter plasmid into the genome of the H2K 2B4 cells without hindering the myogenic characteristics of the cell-line. In addition, the *SB* transposon exhibited a random integration profile with 33% of the integrants being identified in intronic sequences of active genes, an observation consistent to previous studies in other cell-lines [30], [31], [33]. Whilst the integration profile of the *SB* transposon is deemed safer than retroviral vectors, further work is still required to guarantee integration into pre-determined genome sites and thus prevent any potential insertional mutagenesis. Overall, we can confirm the H2K 2B4 clone is readily transfectable using both viral or non-viral transfection methods and that most importantly; genetic modification does not hinder the myogenicity of the line.

Our current findings extend the original work of Morgan and colleagues [8]. In that paper, cells were obtained by enzymatic disaggregation of mouse hindlimb muscles so it was not possible to determine either what muscle the cells were derived from, or if they were indeed (as would be expected) satellite cell-derived. The *in vitro* myogenicity of clones derived and expanded from these muscles was verified, but not quantified and the myogenicity of only one clone was determined on western blot, using an antibody to myogenin. In this study, we prepared cells from isolated fibres of mouse EDL muscles, so we know both the muscle of origin and that the myogenic cells were indeed satellite cell-derived. In addition, we used antibodies to Pax 7, MyoD, myogenin, desmin and myosin to determine the timing and extent of activation, commitment to differentiation, myogenic differentiation and self-renewal of a satellite cell derived cell-line [22]. These data provides a detailed baseline for future users of this clone and can used to determine whether any interventions have had a deleterious effect on the cell's myogenic capacity. The H2K 2B4 cell-line therefore represents a robust reagent for research into muscle cell biology, in contrast to other less well-characterised immortomouse myogenic cell lines [43], [44], [45].

Some of the original *H2K^b^*tsA58 clones developed by Morgan and colleagues, were serially-passaged from one host mouse to another, to show that they had given rise to cells of donor origin that retained their capability of contributing to muscle regeneration [8]. However, it was not determined whether the original grafted cells had given rise to cells in the satellite cell position. In contrast, we have demonstrated here that at least some of our grafted H2KB4 myoblasts gave rise to satellite cells of donor origin.

In summary, this study generated a conditionally immortal satellite cell derived cell-line from the *H-2K^b^*-*tsA58* immortomouse. Extensive *in vitro* and *in vivo* characterisation confirmed the H2K 2B4 clone exhibits muscle stem cell characteristics. The stem cell model could be readily transfected using viral and non-viral techniques. More importantly, using the *SB* system, we have shown the H2K 2B4 cell-line can be genetically modified without hindering the cell's natural characteristics. Overall the H2K 2B4 cell-line combines the myogenic characteristics of satellite cells and the longevity and reliability of a cell-line, making the clone an ideal cell model for muscle research.

## Supporting Information

Figure S1 **Comparison of myogenic protein expression in terminal differentiated in** ***H-2K^b^*** **clones.** Satellite cell derived clones were grown in non permissive conditions (37°C+no γ-IFN) for three days and myogenic protein expression was assessed within the terminally differentiated myotubes. A) Myogenic proteins (MyoD, Slow/Fast myosin and Myogenin) were significantly lower in the H2K 3A4 cell-line compared to the other clones. No significant difference in myogenic protein expression levels was observed between the remaining cell lines. MF20 antibody targets fast & slow myosin. * p<0.05, ** p<0.01, *** p<0.001, One way ANOVA and Tukey test. Error bars generated from SEM, N value of 4.B) Images showing terminally differentiated conditionally immortal clones. Scale bar: 20 microns.
(TIF)

Figure S2 **Terminal differentiation of genetically modified H2K 2B4 cells.** Using Nuclefection, the H2K 2B4 cell-line was transfected with 2.5 µg of the pMONO-neo-eGFP plasmid, 48 hours post transfection transient expression of eGFP protein was detected using flow cytometry. B) Images showing genetically modified H2K 2B4 myotubes formed from H2K 2B4 myoblasts transiently expressing the eGFP gene. Scale bar: 50 microns.
(TIF)

Figure S3 **Analysing Sleeping Beauty integration sites within H2K 2B4 cells using the plasmid recovery method.** A) Schematic diagram of the *Sleeping Beauty (SB)* transposon, pT2/MONO-neo-eGFP. The plasmid contains SB100 transposase binding sites within the inverted terminal repeats (IRDR, purple triangles). The IRDRs flank an eGFP gene, *neo* gene and a ColE1 origin of replication (ori) region. The *neo* gene confers resistance to G418 and kanamycin in mammalian cells and *E.coli*, respectively. The plasmid backbone contains an ampicillin resistance gene and another ColE1 ori region, to distinguish between transposon mediated and random integration when using the plasmid recovery method. B) A diagram to show the two possible integration outcomes of the pT2/MONO-neo-eGFP. Firstly by transposition, a transposase meditated integration, resulting in kanamycin resistance within *E.coli*. Secondly, by random integration resulting in resistance to both kanamycin and ampicillin antibiotics. C) A brief outline of the plasmid recovery method.
(TIF)
