# Supplementary figures and images for: A New Extensively Characterised Conditionally Immortal Muscle Cell-Line for Investigating Therapeutic Strategies in Muscular Dystrophies
Source: PLoS One. 2011 Sep 14;6(9):e24826. doi: 10.1371/journal.pone.0024826 (PMC3173493; doi:10.1371/journal.pone.0024826)

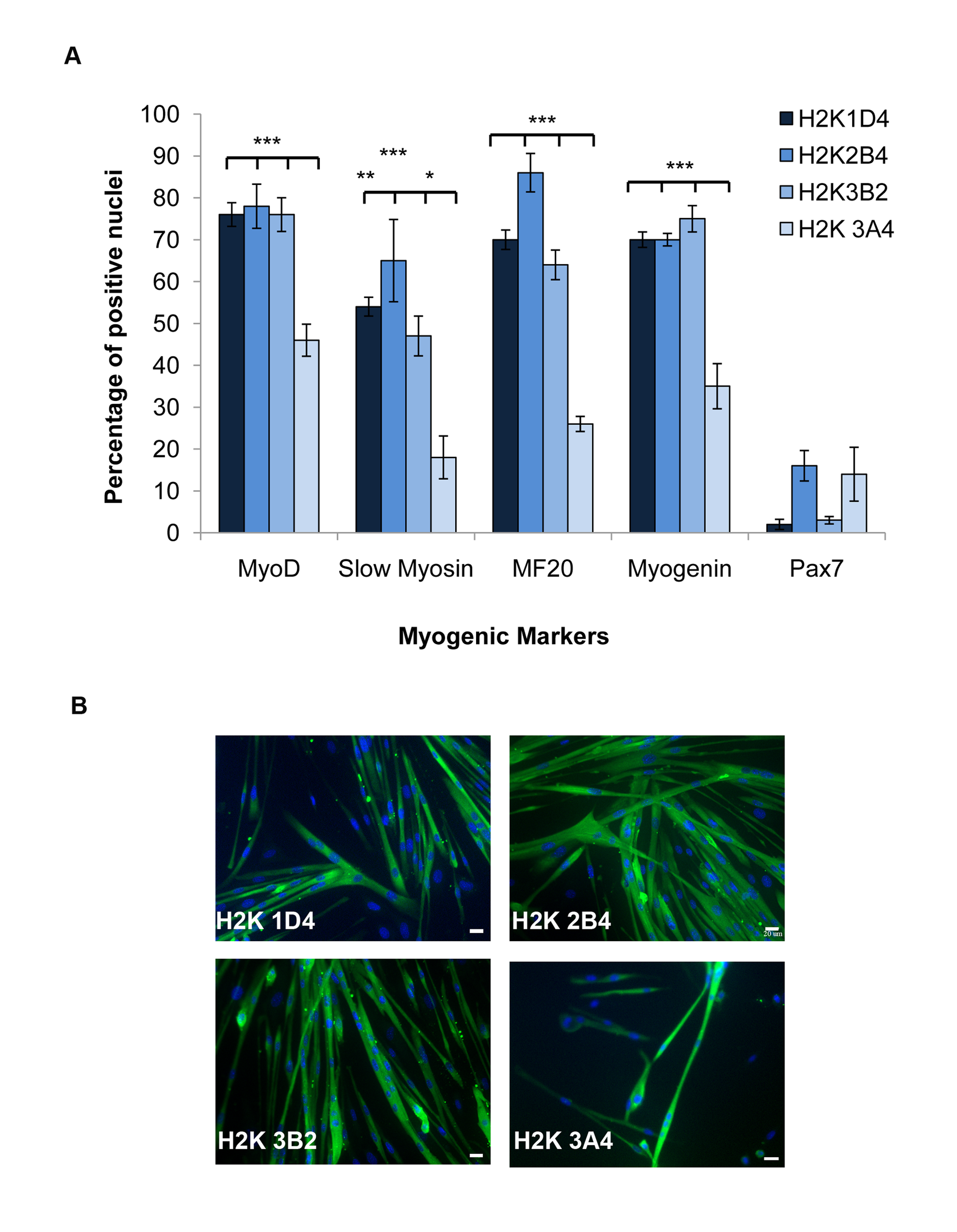

Supplement: Figure S1 — Comparison of myogenic protein expression in terminal differentiated in H-2Kb clones. Satellite cell derived clones were grown in non permissive conditions (37°C+no γ-IFN) for three days and myogenic protein expression was assessed within the terminally differentiated myotubes. A) Myogenic proteins (MyoD, Slow/Fast myosin and Myogenin) were significantly lower in the H2K 3A4 cell-line compared to the other clones. No significant difference in myogenic protein expression levels was observed between the remaining cell lines. MF20 antibody targets fast & slow myosin. * p<0.05, ** p<0.01, *** p<0.001, One way ANOVA and Tukey test. Error bars generated from SEM, N value of 4.B) Images showing terminally differentiated conditionally immortal clones. Scale bar: 20 microns. (TIF) [file pone.0024826.s001.tif]

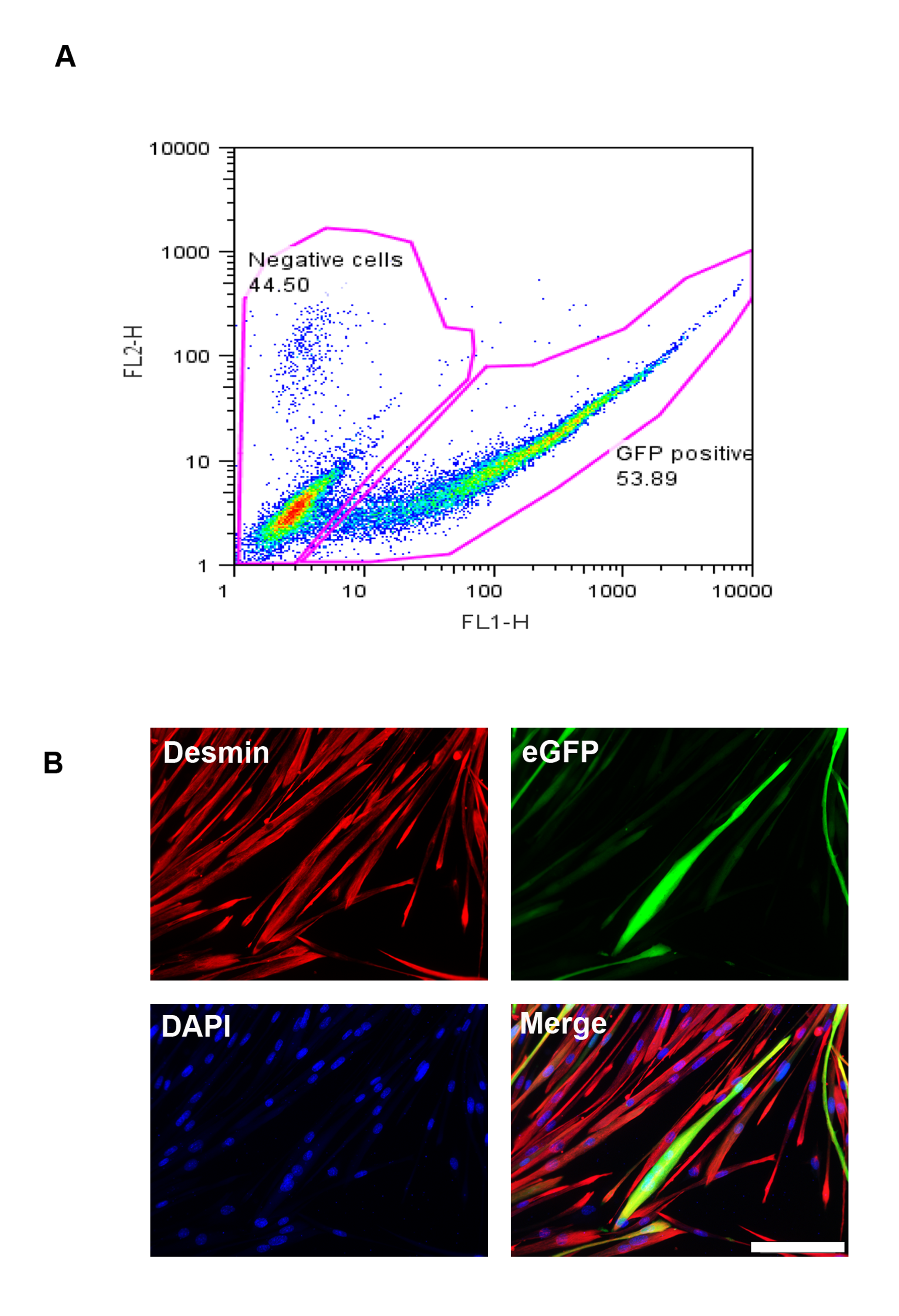

Supplement: Figure S2 — Terminal differentiation of genetically modified H2K 2B4 cells. Using Nuclefection, the H2K 2B4 cell-line was transfected with 2.5 µg of the pMONO-neo-eGFP plasmid, 48 hours post transfection transient expression of eGFP protein was detected using flow cytometry. B) Images showing genetically modified H2K 2B4 myotubes formed from H2K 2B4 myoblasts transiently expressing the eGFP gene. Scale bar: 50 microns. (TIF) [file pone.0024826.s002.tif]

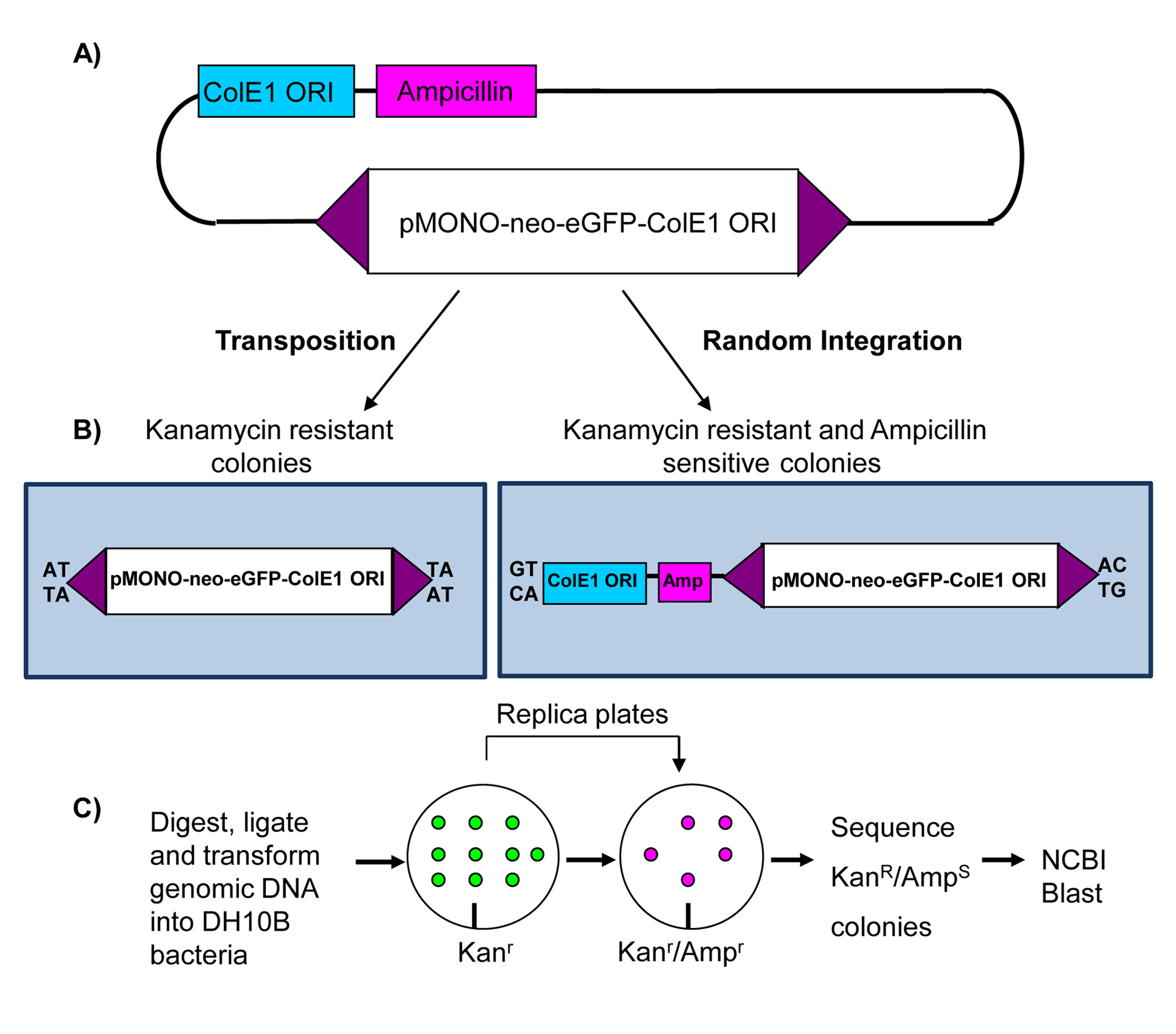

Supplement: Figure S3 — Analysing Sleeping Beauty integration sites within H2K 2B4 cells using the plasmid recovery method. A) Schematic diagram of the Sleeping Beauty (SB) transposon, pT2/MONO-neo-eGFP. The plasmid contains SB100 transposase binding sites within the inverted terminal repeats (IRDR, purple triangles). The IRDRs flank an eGFP gene, neo gene and a ColE1 origin of replication (ori) region. The neo gene confers resistance to G418 and kanamycin in mammalian cells and E.coli, respectively. The plasmid backbone contains an ampicillin resistance gene and another ColE1 ori region, to distinguish between transposon mediated and random integration when using the plasmid recovery method. B) A diagram to show the two possible integration outcomes of the pT2/MONO-neo-eGFP. Firstly by transposition, a transposase meditated integration, resulting in kanamycin resistance within E.coli. Secondly, by random integration resulting in resistance to both kanamycin and ampicillin antibiotics. C) A brief outline of the plasmid recovery method. (TIF) [file pone.0024826.s003.tif]
